# An Immune-Related Long Noncoding RNA Pair as a New Biomarker to Predict the Prognosis of Patients in Breast Cancer

**DOI:** 10.3389/fgene.2022.895200

**Published:** 2022-06-22

**Authors:** Hanwen Jiang, Jingxian Sun, Fucong Liu, Xincai Wu, Zhaohui Wen

**Affiliations:** ^1^ Department of Neurology, Brain Ultrasound, The First Affiliated Hospital of Harbin Medical University, Harbin, China; ^2^ Department of Neurosurgery, The First Affiliated Hospital of Harbin Medical University, Harbin, China

**Keywords:** breast cancer, immune-related lncRNA pair, MIR4435-2HG, biomarker, TCGA

## Abstract

**Background:** Immune-related long non-coding RNAs (irlncRNAs) might remodel the tumor immune microenvironment by changing the inherent properties of tumor cells and the expression of immune genes, which have been used to predict the efficacy of immunotherapy and the prognosis of various tumors. However, the value of irlncRNAs in breast cancer (BRCA) remains unclear.

**Materials and Methods:** Initially, transcriptome data and immune-related gene sets were downloaded from The Cancer Genome Atlas (TCGA) database. The irlncRNAs were extracted from the Immunology Database and Analysis Portal (ImmPort) database. Differently expressed irlncRNAs (DEirlncRNAs) were further identified by utilizing the limma R package. Then, univariate and multivariate Cox regression analyses were conducted to select the DEirlncRNAs associated with the prognosis of BRCA patients. In addition, the univariate and least absolute shrinkage and selection operator (LASSO) Cox regression analyses were performed to determine the DEirlncRNA pairs with the independent prediction capability of prognosis in BRCA patients. Finally, the chosen DEirlncRNA pair would be evaluated in terms of survival time, clinicopathological characteristics, tumor-infiltrating immune cells, immune checkpoints (ICs), signaling pathways, and potential small-molecule drugs.

**Results:** A total of 21 DEirlncRNA pairs were extracted, and among them, lncRNA MIR4435-2HG and lncRNA U62317.1 were chosen to establish a risk signature that served as an independent prognostic biomarker in BRCA patients. Patients in the high-risk group had a worse prognosis than those in the low-risk group, and they also had an abundance of infiltration of CD4^+^ T and CD8^+^ T cells to enhance the immune response to tumor cells. Furthermore, the risk signature showed a strong correlation with ICs, signaling pathways, and potential small-molecule drugs.

**Conclusion:** Our research revealed that the risk signature independent of specific DEirlncRNA pair expression was closely associated with the prognosis and tumor immune microenvironment in BRCA patients and had the potential to function as an independent prognostic biomarker and a predictor of immunotherapy for BRCA patients, which would provide new insights for BRCA accurate treatment.

## Introduction

BRCA is the most common malignant tumor in women, with the highest morbidity among female malignancies worldwide (Siegel et al., 2021). BRCA, an extremely complex cancer type with quite strong molecular heterogeneity (Ahn et al., 2021), which has a high recurrence rate and mortality rate, has become a great threat to the health of women around the world (Saatci et al., 2021). Although the therapeutic effect of BRCA has made great strides, regretfully, there is still a lack of pragmatic markers and diagnostic methods for predicting the prognosis of BRCA patients (Pupa et al., 2021). According to the cause of tumor formation, BRCA could be classified into four subtypes on the molecular level (i.e., Luminal A, Luminal B, Her-2 positive, and triple-negative BRCA) (Okines and Turner, 2021), whereas the prognosis and treatment outcomes of BRCA patients with the same molecular subtypes still vary greatly. In recent years, the emergence of tumor immunotherapy has brought about a new change for BRCA clinical treatment, which is related to the activation and development of immunocytes in the tumor microenvironment (TME) (Song H. N. et al., 2021). Therefore, it is essential for personalized BRCA treatment to find influential molecular makers, evaluate the BRCA tumor immunoreactivity and establish convincing prognostic models.

TME is an extremely intricate network of internal environment, which was composed of tumor stromal cells and active factors secreted by them, vascular and lymphatic nets, and the extracellular matrix (Xiang et al., 2022). Immunocytes and stromal cells are the most common non-tumor cells in the TME. It has been proved that autologous tumor-infiltrating lymphocytes (TILs) and IC inhibitors administration could mediate tumor progression by targeting immunogenic tumor mutations (Bu et al., 2021). Although BRCA was originally classified as a low immunogenic tumor, BRCA with worse prognostic characteristics still showed an immunogenic TME with more TILs (Sorrentino et al., 2021). For example, programmed cell death ligand 1 (PD-L1) and low levels of TILs were both associated with poor clinical prognosis in BRCA patients, which provides a scientific basis for the use of immune checkpoint blockade (ICB) therapy in BRCA patients (Zhu et al., 2019). Similarly, different TIL subtypes (CD4^+^ TILs, CD8^+^ TILs, and FOXP3+ TILs) were all correlated with a good prognosis for BRCA (Lotfinejad et al., 2020). Furthermore, it has been confirmed that Delta-like 1 (DLL1) could promote immunotherapy of BRCA by regulating CD8^+^ T cells to keep TME in a normal state for a long time (Zhang et al., 2021); CD2 expression is related to a variety of tumor-infiltrating immune cells and serves as an immune-associated prognostic biomarker to regulate the TME of BRCA (Chen Y. et al., 2021); ATP2C2 could maintain the immune dominance of TME, which might serve as a prognostic marker for BRCA patients and provide a potential target for the treatment of BRCA (Liu J. et al., 2021). All of the above could provide help in enhancing the understanding of the tumor immune microenvironment and BRCA-related immune genes. They further demonstrated that BRCA-related immune long non-coding RNAs (lncRNAs) analysis could help to reflect the tumor immune microenvironment of BRCA patients more comprehensively in order to explore molecular markers for effective targeted therapy of BRCA.

LncRNAs with transcripts longer than 200 nucleotides are transcription products of RNA polymerase II (Rey et al., 2021). LncRNA encoding genes are widely distributed in introns or exons of mRNA encoding genes (Lu et al., 2021). Most lncRNAs lack the capacity to be translated into proteins, but they could regulate gene expression through various mechanisms (Yang et al., 2022). Indeed, lncRNAs play an important role in presenting relevant tumor prognosis (Xu et al., 2021). Meanwhile, some studies have shown that tumor-related lncRNAs could also participate in changing the inherent properties of tumor cells and the expression of immune genes to remodel the tumor immune microenvironment (Eptaminitaki et al., 2021). For example, LINC0187 could affect the prognosis of endometrial cancer, whose high expression may indicate a better prognosis (Wang Z. et al., 2021); the immune-autophagy-related lncRNA signatures including MIR210HG, AC09985.3 and CYTOR as unfavorable prognostic determinants could exhibit a predictive ability in hepatocellular carcinoma (HCC) (Wang Y. et al., 2021); lncRNA including major histocompatibility complex-I (MHC-I) and immunogenicity of tumor (LIMIT) could boost guanylate-binding proteins (GBPs) and MHC-1 to enhance tumor immunogenicity and checkpoint therapy, and the LIMIT-GBP-heat shock factor-1 (HSF1) axis might be a target of cancer immunotherapy (Li G. et al., 2021). However, there are few studies on the association between lncRNAs and BRCA immune microenvironment, and the prognostic value of irlncRNAs in BRCA remains unclear recently.

In view of the influence and regulation of the tumor immune microenvironment and lncRNAs on BRCA, we aimed to identify the lncRNAs associated with immunity to BRCA and explore the value of irlncRNAs in the TME and prognosis of BRCA. Initially, TCGA-BRCA public database was searched to explore the irlncRNAs, and the DEirlncRNAs were determined by limma R package and Cox risk regression models to interpret lncRNAs with diagnostic and prognostic-predicting ability. A prognostic risk signature was then constructed by creating an innovative lncRNA pairing signature. The pair (MIR4435-2HG|U62317.1) was found to have great significance in BRCA, not only with diagnostic and prognostic ability for BRCA patients, but also with the ability to predict potential small-molecule drugs and assess the immune cell infiltration in the TME of BRCA. Ultimately, our results indicated the significance of the risk signature in the TME and provided new insights for the effective treatment of BRCA.

## Materials and Methods

### Data Acquisition and Integration

The transcriptome data and the corresponding clinical data of 1,109 BRCA samples and 113 normal tissue samples were downloaded and integrated from the TCGA database (https://portal.gdc.cancer.gov/), which could verify the clinical characteristics and prognostic value of lncRNAs (Sun et al., 2021). Immune-related gene sets were obtained from the ImmPort database (http://www.immport.org) for the difference and co-expression analysis (Yin et al., 2021), while irlncRNAs could be determined through the Pearson correlation analysis of expression levels between lncRNAs and immune-related genes according to the correlation coefficients >0.5 and *p* < 0.001.

### Differential Expression Analysis of Immune-Related Genes

Based on the data acquired above, irlncRNAs were screened from the TCGA-BRCA database, and the limma R package (version 3.6.3, https://www.r-project.org) was used to perform the differential expression analysis among irlncRNAs (Song S. et al., 2021), aiming to select DEirlncRNAs with the criteria of |log Fold Change (logFC)| ≥ 1.5 and *P* < 0.05. Then, univariate Cox regression analysis was conducted based on the clinical survival time to extract DEirlncRNAs with significance, using *p* < 0.01 as an identifying threshold. Following that, multivariate Cox regression analysis was performed to determine the DEirlncRNAs associated with the prognosis of BRCA patients.

### Determination of DEirlncRNA Pairs

The DEirlncRNAs with the TCGA-BRCA risk prediction capability from the prior risk signature was further used to establish a DEirlncRNA pair model. And resulting DEirlncRNA pairs were presented as a 0-or-1 matrix, in which 1 represents that a higher expression was exhibited in lncRNA A compared to lncRNA B, while 0 represents the opposite. Thereafter, the constructed 0-or-1 matrix was further evaluated. When the ratio of DEirlncRNA pairs assessed as 0 or one accounted for more than 80% of all pairs acquired, it meant that these pairs were not related to the prognosis of BRCA due to the lack of a definite grade that could appropriately predict the survival outcome of patients (Liu Y. et al., 2021). Therefore, DEirlncRNA pairs that were valued at 0 or 1 with a proportion of 20–80% of the total pairs could predict the prognosis of BRCA effectively, which will be ulteriorly investigated.

### Construction and Validation of the Risk Signature

Initially, univariate Cox regression analysis was performed to depict the prognostic DEirlncRNA pairs. Next, the LASSO Cox regression analysis that has the ability to avoid over-fitting, was applied to establish an optimal risk model for the independent prognostic DEirlncRNA pairs based on the glmnet R package. After that, the Kaplan-Meier survival analysis was performed to evaluate the difference in overall survival (OS) between the high-risk group and low-risk group patients, which could verify the prognostic value of the risk signatures for each BRCA patient utilizing the survival and survminer package in R (Huang et al., 2021). Then, the risk level of each patient was graded, in which the riskScore of high-risk patients was set as one and the opposite was set as 0. Further, clinicopathological characteristics (tumor stage, and T, N, and M stages) of the patients were evaluated by univariate and multivariate Cox regression analysis to demonstrate the ability of this pair to predict the clinical prognosis independently. *p* < 0.05 indicated a statistically significant difference.

### Investigation of Tumor-Infiltrating Immune Cells

The immune cell infiltration data of total samples from the TCGA-BRCA database was employed to analyze the association between the risk signature and tumor-infiltrating immune cells based on the techniques including XCELL, TIMER, QUANTISEQ, MCPCOUNTER, EPIC, CIBERSORT-ABS, and CIBERSORT (Wang et al., 2019). After analyzing the contents of infiltrating immune cells, the Wilcoxon signed-rank test was used to evaluate the differences between the high-risk and low-risk groups, designed by MIR4435-2HG|U62317.1, with regard to tumor-infiltrating immune cells, utilizing a boxplot to depict (Li C. et al., 2021). And Spearman correlation analysis was used to evaluate the relationship between the riskScore and the tumor-infiltrating immune cells, utilizing a lollipop chart to demonstrate the resulting correlation coefficient (Gu et al., 2021). This procedure was carried out with the ggplot2 package in R and *p* < 0.05 as an identifying threshold. Furthermore, the differences in various types of tumor-infiltrating immune cells in the model groups were also detected according to the construction of our model.

### Recognition of Drug Molecules

To evaluate the effect of potential small-molecule drugs on the treatment of BRCA by DEirlncRNA pair, the connectivity map (CMap) database (http://clue.io/) was utilized to analyze the data of the genes with the co-expression of the two lncRNAs in the established risk signature. In addition, CMap connectivity score > 90 or < -90 was considered as the criteria for identifying effective small-molecule drugs.

### Statistical Analysis

The above statistical investigations were entirely carried out by taking advantage of bioinformatics databases and tools available online. R software version 3.6.1 was used for comprehensive statistical analyses. When the data was distributed regularly, the means and medians of continuous variables were compared using the Student’s t-test; otherwise, the Wilcoxon test was employed. The Pearson correlation analysis was conducted to determine the connection between two variables. The Kaplan-Meier analysis along with the long-rank test was used to assess differences in OS between the two risk groups. And univariate and multivariate Cox regression were used to analyze the resulting data with the independent parameters associated with being bilateral for all tests, and *p* < 0.05 was statistically significant.

## Results

### Identification of DEirlncRNAs With Prognostic Value in BRCA

**FIGURE 1:**
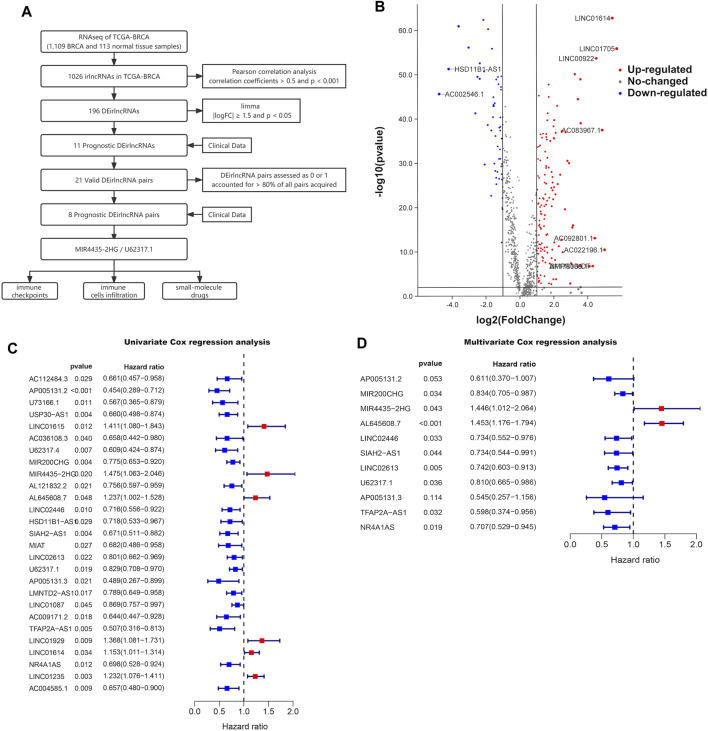
Prognostic value in BRCA. **(A)** Workflow of this study. **(B)** Volcano plot of differentially expressed irlncRNAs between 113 normal and 1,109 tumor tissue samples in the TCGA-BRCA cohort. Forest plots display the differential expression of irlncRNAs associated with OS. HR and 95% CI were calculated by univariate **(C)** and multivariate **(D)** cox regression analysis.

The procedure of this study is outlined in Figure 1A. We downloaded transcriptome data from the TCGA-BRCA database, which contained 1,109 BRCA samples and 113 normal tissue samples, as well as immune-related gene sets from the ImmPort database. Through the Pearson correlation analysis of lncRNAs and immune-related genes, 1,026 irlncRNAs were determined with correlation coefficients >0.5 and *p* < 0.001, from which 196 irlncRNAs were identified as differentially expressed in the TCGA-BRCA database visualized on the irlncRNA heatmap (Supplemental Figure S1) and volcano plot (Figure 1B), utilizing the limma R package with the criteria of |logFC| ≥ 1.5 and *P* < 0.05. Among them, 52 DEirlncRNAs were lowly expressed, and 144 DEirlncRNAs were highly expressed in BRCA patients. Then, we established univariate and multivariate Cox regression models to recognize the correlation between these DEirlncRNAs and the OS of BRCA patients utilizing the clinical data from TCGA-BRCA, and discovered that a total of 11 DEirlncRNAs were associated with the prognosis of BRCA patients (Figures 1C, D).

### Filtration of DEirlncRNA Pairs With Prognostic Value and Establishment of a Risk Signature

**FIGURE 2:**
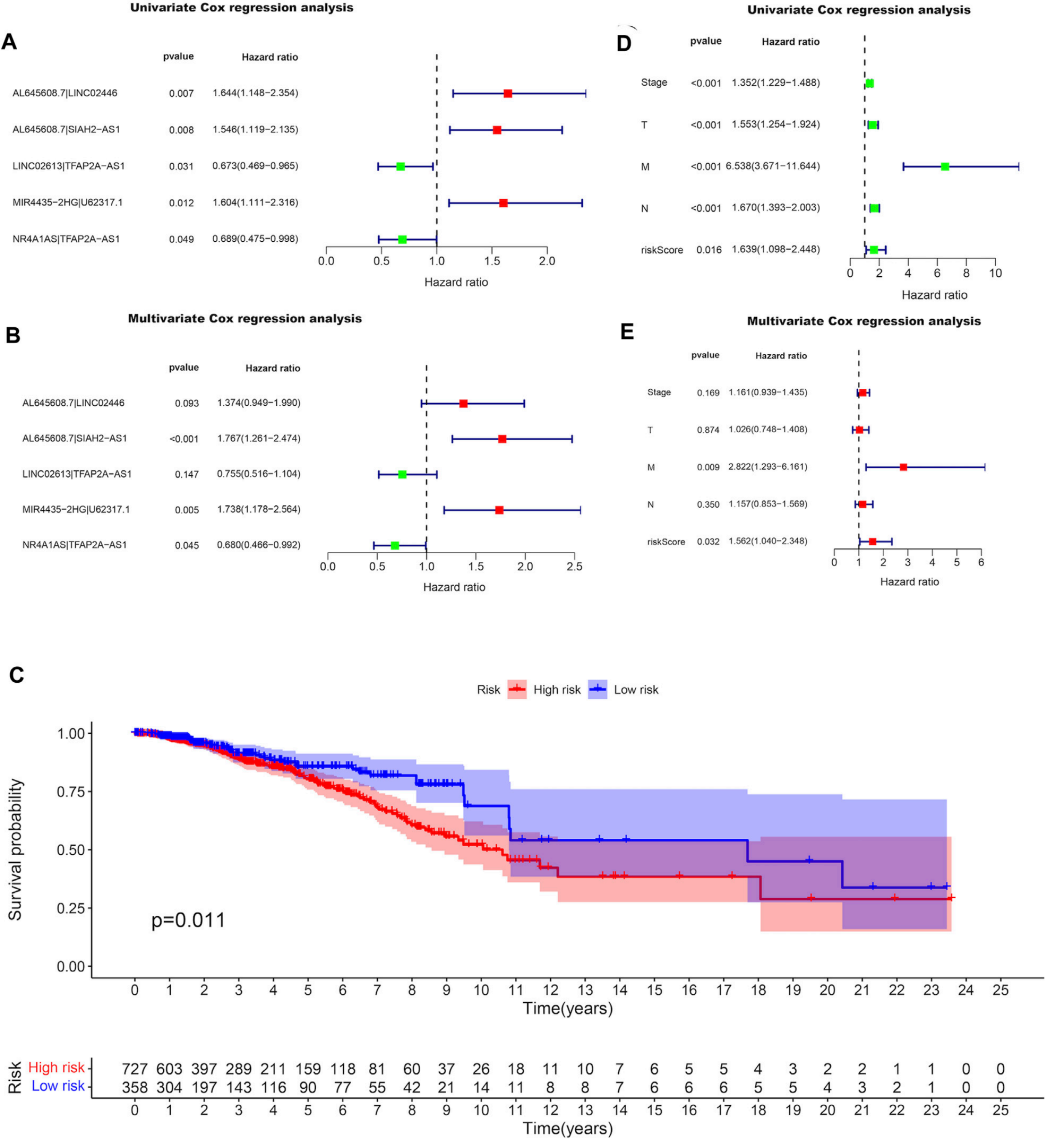
Differentially expression of irlncRNA pairs with prognostic value in BRCA. Forest plots display the differential expression of irlncRNAs pairs associated with OS. HR and 95% CI were calculated by the LASSO univariate **(A)** and multivariate **(B)** cox regression analysis. **(C)** Kaplan-Meier survival curves of the high-risk group and the low-risk group defined with the expression of lncRNA MIR4435-2HG and lncRNA U62317.1. Forest plots display that the univariate **(D)** and multivariate **(E)** cox regression analyses were performed for the analysis of clinicopathological features with the pair MIR4435-2HG|U62317.1.

By pairing the resulting 11 DEirlncRNAs with the TCGA-BRCA risk prediction capability, which was evaluated by a 0-or-1 matrix, we selected eight DEirlncRNA pairs with prognostic value among the obtained 21 DEirlncRNA pairs using univariate Cox regression analysis (Supplementary Table S1). After matching the clinical data of BRCA patients with the constructed DEirlncRNA pairs, the DEirlncRNA pairs with the independent prediction capability of prognosis in BRCA patients were determined through the LASSO univariate and multivariate Cox regression analyses successively **(** Figures 2A, B; Supplementary Figure S2). The paired DEirlncRNAs obtained above were screened to extract the DEirlncRNA pairs coupled by two DEirlncRNAs with the same expression trend, and since only lncRNA MIR4435-2HG had significance on the Kaplan-Meier survival analysis among the extracted DEirlncRNA pairs (Supplementary Figure S3), we chose the DEirlncRNA pair of lncRNA MIR4435-2HG and U62317.1 to establish a risk signature.

### Assessment of the Prognostic Value of the Risk Signature

The expressions of lncRNA MIR4435-2HG and lncRNA U62317.1 were compared in the established risk signature, in which a higher expression of lncRNA U62317.1 compared to lncRNA MIR4435-2HG was defined as the low-risk group; otherwise, it was defined as the high-risk group. After performing the Kaplan-Meier survival analysis to evaluate the differences in OS between the two groups, we learned that the survival time of the patients in the high-risk group was notably shorter than that of the patients in the low-risk group (*P* = 0.011) (Figure 2C). Furthermore, univariate and multivariate Cox regression analyses were conducted to assess the riskScore and the clinicopathological characteristics of high-risk and low-risk patients. The data was visualized on forest maps using the survival package in R. Among them, the results of univariate Cox regression analysis demonstrated that there were significant differences in the riskScore of the irlncRNA pair (hazard ratio [HR], 1.639; 95% confidence interval [CI], 1.098-2.448; *P* = 0.016) and the clinicopathological characteristics (Figure 2D), while the results of multivariate Cox regression analysis indicated that differences in the riskScore of the irlncRNA pair that served as a prognostic marker could independently predict the prognosis of BRCA patients (HR, 1.562; 95% CI, 1.040-2.348; *P* = 0.032), and metastasis status was also an independent predictor (HR, 2.822; 95% CI, 1.293-6.161; *P* = 0.009) (Figure 2E).

### Association of the Risk Signature With Immune Characteristics

**FIGURE 3:**
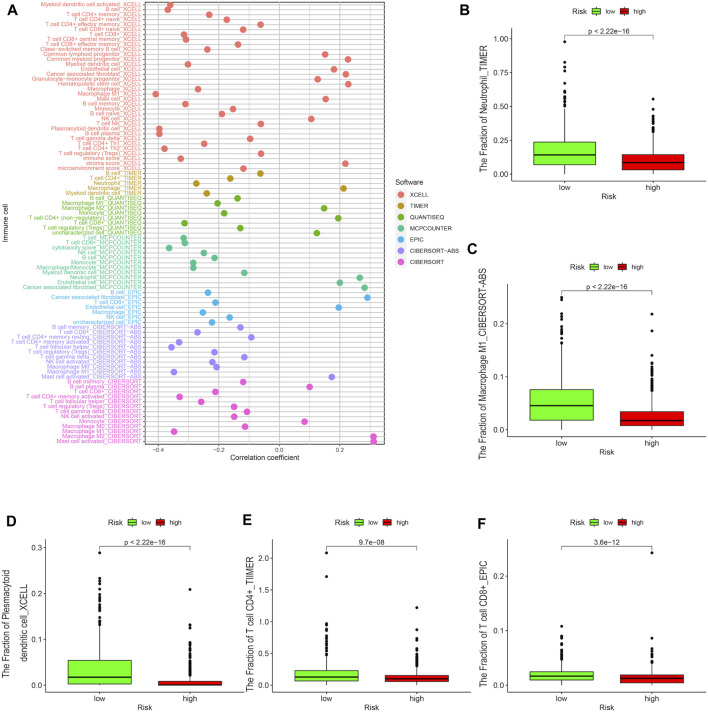
Association of the risk signature with immune characteristics. **(A)** The Wilcoxon signed-rank test was performed to evaluate the correlation between the high-risk and low-risk groups concerning tumor-infiltrating immune cells. The high-risk group was negatively correlated with the fraction of tumor-infiltrating immune cells, such as neutrophils **(B)**, M1 macrophages **(C)**, plasmacytoid dendritic cells **(D)**, CD4^+^ T cells **(E)**, and CD8^+^ T cells **(F)**.

**FIGURE 4:**
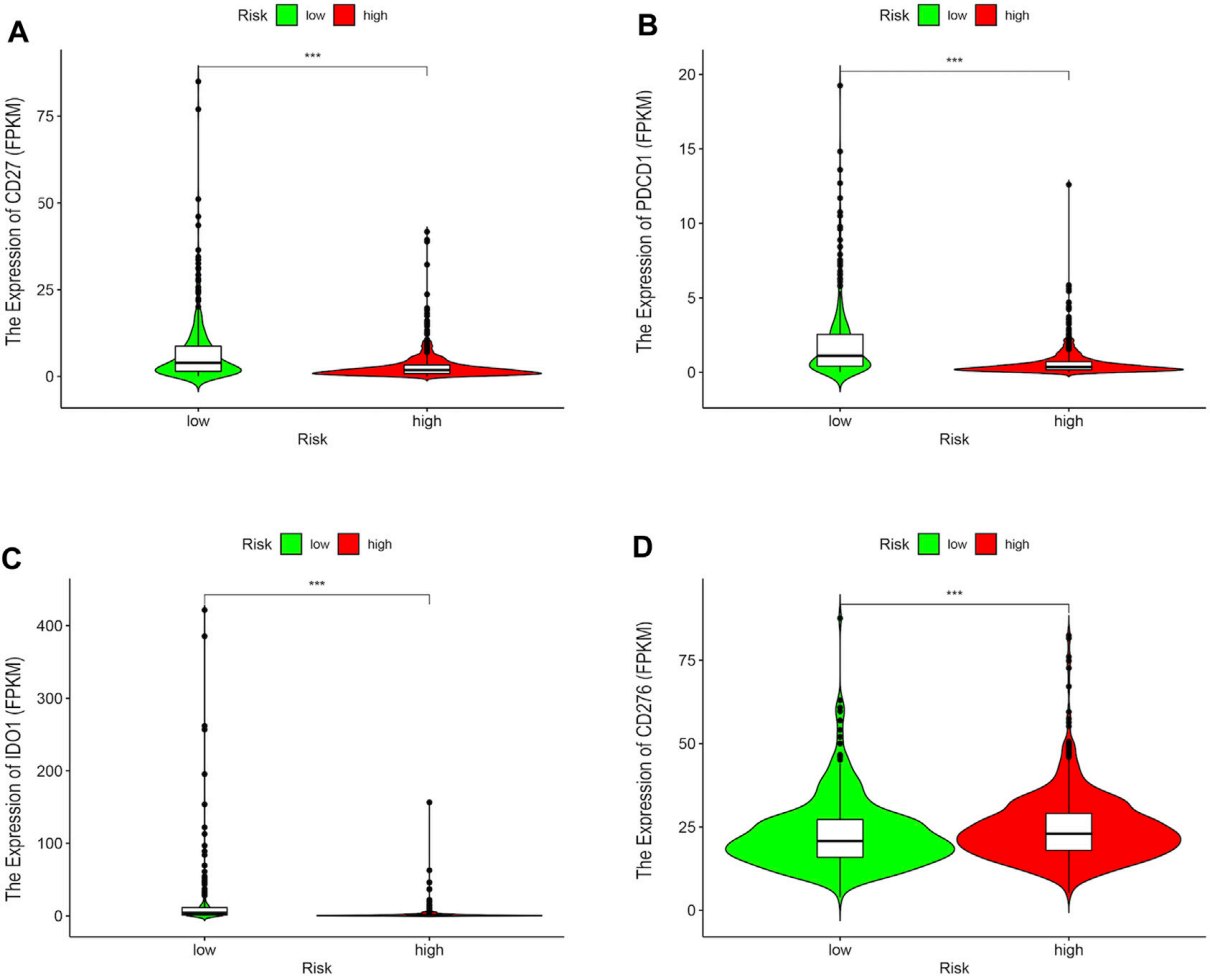
The correlation between the risk signature and the expressions of several ICs. The high-risk group exhibited lower expressions of CD27 **(A)**, PCDC1 **(B)**, and IDO1 **(C)**, and higher expression of CD276 **(D)** than those in the low-risk group.

Since the DEirlncRNA pairs were initially extracted from immune-related genes, we further investigated the correlation between this riskScore and immune characteristics. The Wilcoxon signed-rank test was performed to evaluate the differences between the high-risk and low-risk groups with regard to tumor-infiltrating immune cells (Figure 3A). The findings indicated that the high-risk group was negatively correlated with tumor-infiltrating immune cells, such as neutrophils, M1 macrophages, plasmacytoid dendritic cells, CD4^+^ T cells, and CD8^+^ T cells (Figures 3B–F), whereas it was positively correlated with cancer-associated fibroblasts and M2 macrophages (Supplementary Figure S4). Meanwhile, the results of Spearman correlation analysis that was conducted to assess the data based on the techniques including XCELL, TIMER, QUANTISEQ, MCPCOUNTER, EPIC, CIBERSORT-ABS, and CIBERSORT revealed that the high-risk patients had lower immune and microenvironment scores, which were depicted on a lollipop chart. Furthermore, the correlation between the riskScore and the expressions of several ICs that play important roles in the treatment of BRCA was evaluated. It was found that patients in the high-risk group exhibited lower expression of CD27, PCDC1, and IDO1, and higher expression of CD276 than those in the low-risk group (Figure 4). All these results suggested that the selected DEirlncRNA pair was bound up with tumor-infiltrating immune cells, the immune microenvironment, and the expressions of related ICs, which could predict the efficacy of immunotherapy for BRCA patients.

### Association of a Single Paired-Gene With BRCA

**FIGURE 5:**
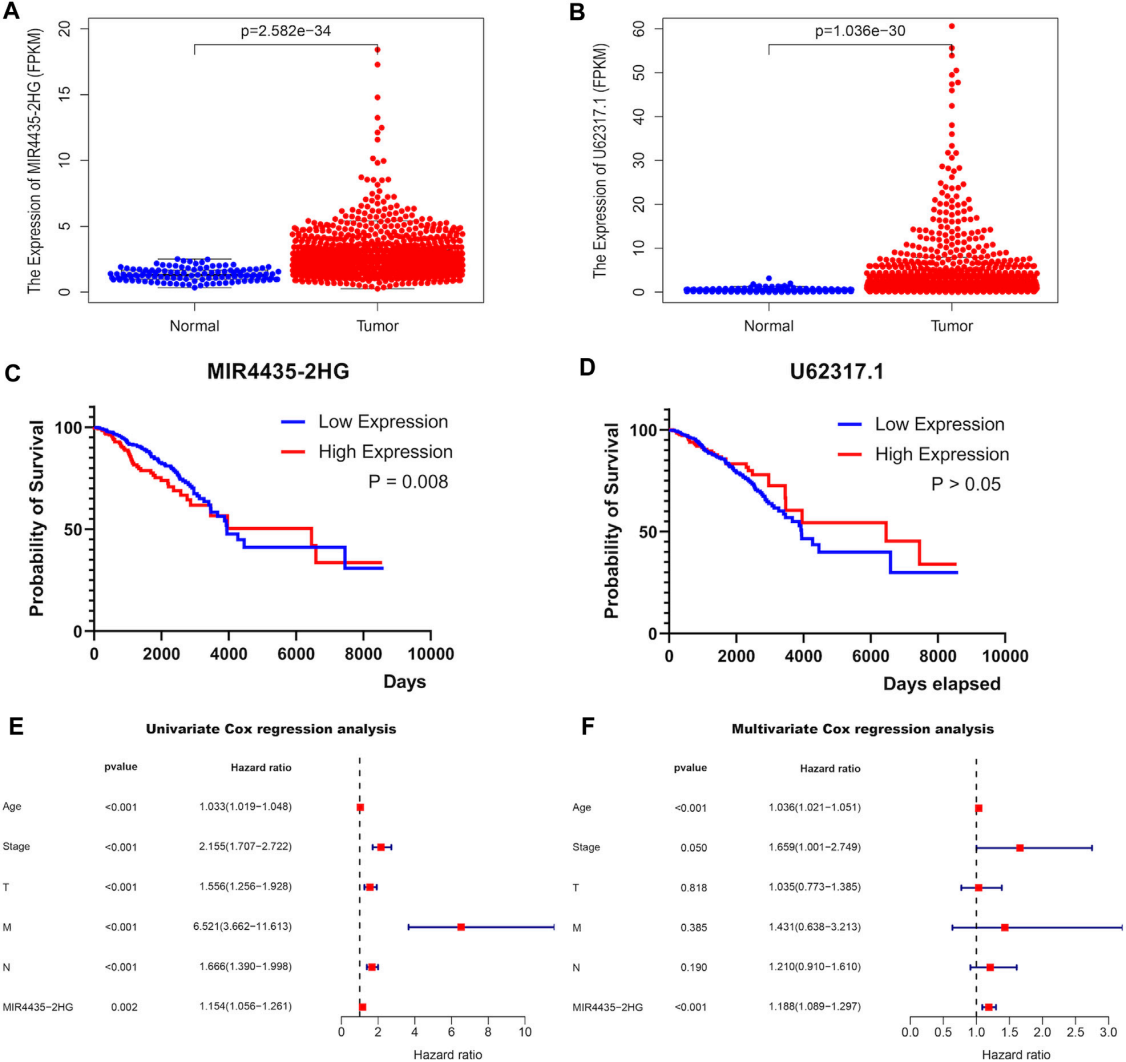
Association of a single paired-DEirlncRNA with TCGA-BRCA. The expressions of lncRNA MIR4435-2HG **(A)** and lncRNA U62317.1 **(B)** between 113 normal and 1,109 tumor tissue samples in the TCGA-BRCA cohort. Kaplan-Meier survival curves of lncRNA MIR4435-2HG **(C)** and lncRNA U62317.1 **(D)**. Forest plots display that the univariate **(E)** and multivariate **(F)** cox regression analyses were performed the analysis of clinicopathological features with the expression of MIR4435-2HG.

**FIGURE 6:**
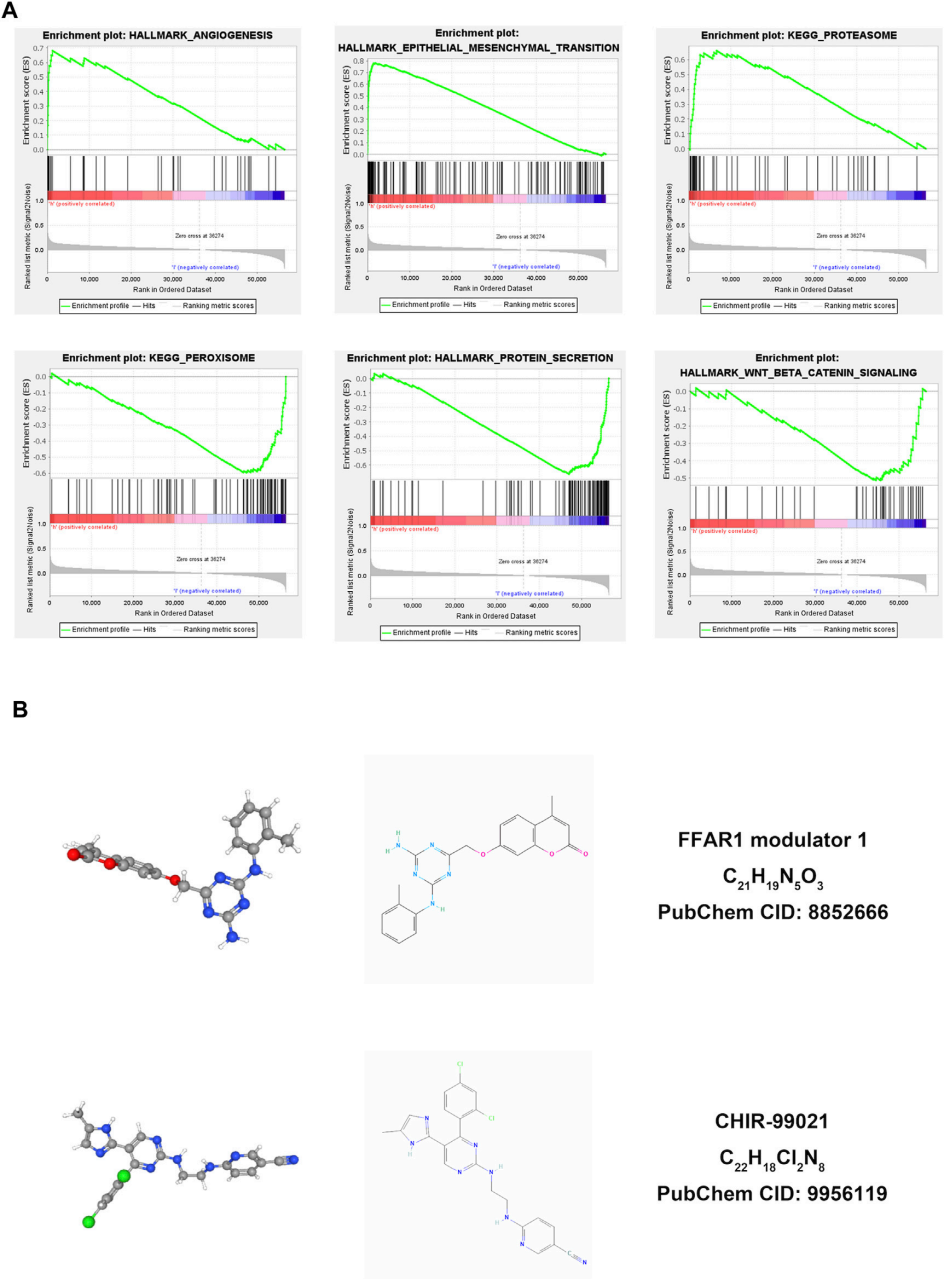
The GSEA results with the expression of MIR4435-2HG and prediction of small-molecule drugs for BRCA. **(A)** The high expression group of MIR4435-2HG was primarily enriched in some signaling pathways, such as KEGG-Proteasome, KEGG-EMC RECEPTOR INTERACTION, HALLMARK EMT, HALLMARK ANGIOGENESIS, and GOBP_REGULATION_OF_EXTRACELLULAR_MATRIX_ORGANIZATION. **(B)** The 2D and 3D structures of these two compounds were identified by the connectivity map analysis: FFAR1 modulator 1 (High) and CHIR-99021 (Low).

After investigating immune characteristics on BRCA patients in the risk signature established by pairing two DEirlncRNAs, we continued to analyze the correlation between the two lncRNAs and BRCA separately. Initially, the expressions of lncRNA MIR4435-2HG (Figure 5A) and lncRNA U62317.1 (Figure 5B) in BRCA patients were evaluated to discover that both of them were highly expressed. After performing the Kaplan-Meier survival analysis to evaluate the differences in OS between the two lncRNAs and BRCA, whose expression levels were divided by the cut-off point of the median expression of the corresponding DEirlncRNA, we learned that patients with the higher expression of lncRNA MIR4435-2HG had a shorter survival time (Figure 5C), but lncRNA U62317.1 showed no significant difference in survival time (Figure 5D). Therefore, lncRNA MIR4435-2HG with differential survival in OS was analyzed in combination with clinical information. Univariate (Figure 5E) and multivariate (Figure 5F) Cox regression analyses were conducted to show that lncRNA MIR4435-2HG was an independent predictor for BRCA patients. In addition, comparing the expressions of lncRNA MIR4435-2HG in BRCA patients, the patients with the expression levels of lncRNA MIR4435-2HG in the top 50% were in the high expression group, while the other patients were in the low expression group. The above two groups were subjected to gene set enrichment analysis (GSEA), which revealed that the high expression group was primarily enriched in some signaling pathways, such as KEGG-Proteasome, KEGG-EMC RECEPTOR INTERACTION, HALLMARK EMT, HALLMARK ANGIOGENESIS, and GOBP_REGULATION_OF_EXTRACELLULAR_MATRIX_ORGANIZATION, and these also provided downstream targets for further research of lncRNA MIR4435-2HG (Figure 6A).

### Prediction of Small-Molecule Drugs for BRCA

Because the expressions of both lncRNA MIR4435-2HG and lncRNA U62317.1 in the established risk signature were upregulated in BRCA patients, we identified 10 genes with the co-expression of the two lncRNAs, using the Pearson correlation analysis with correlation coefficients >0.5 and *p* < 0.001. The 10 genes were uploaded to the CMap website in order to predict the potential small-molecular drugs for BRCA, and two small-molecular compounds were obtained based on the screening criteria. The 2D and 3D structures of these two compounds were depicted on a schematic diagram in Figure 6B.

## Discussion

In recent decades, there has been a great improvement in OS of BRCA patients, but metastasis or death still occurs in 15% of patients (Bertucci and Goncalves, 2017). Due to the high heterogeneity of BRCA, the effects of postoperative chemotherapy and endocrine therapy are significantly different (Subhan and Muzibur Rahman, 2022). Although it is traditionally considered that the immunogenicity of BRCA is poor compared with melanoma (Kalaora et al., 2022) and renal cell carcinoma (Zhou et al., 2022), which have made enormous progress in immunotherapy, the latest studies suggest that immunotherapy may activate immune activation pathways and specifically kill tumor cells, providing a new way to further improve the prognosis of BRCA patients (Adeshakin et al., 2022). Therefore, it is crucial for personalized BRCA treatment to explore a unique prognostic risk signature for the preliminary evaluation of the efficacy of immunotherapy and small-molecular drug therapy and the possible prognosis. By far, many tumor-related lncRNAs have been used as biomarkers to predict the prognosis of multiple tumors, based on their ability to change the inherent properties of tumor cells (Najafi et al., 2021). For instance, it has been verified that DLX6-AS1 hypermethylation could be a biomarker for sporadic colorectal cancer (CRC) progression and prognosis, whose high expression might indicate a shorter survival time in CRC patients (Lin et al., 2021). RUSC1-AS1, LINC02609, and SNHG17 exhibited the capability to predict the prognosis of renal cancer, and their expression levels were remarkably higher in patients with high-risk (Shu et al., 2021). Besides, colon cancer-associated transcript 2 (CCAT2) could be valuable to monitor the prognosis of cervical cancer (CC) patients, and it will improve the diagnostic efficiency of CC with the combination of CCAT2 (Cao et al., 2022). However, previous studies developed signatures primarily using the specific expression levels of transcriptome data (Najafi et al., 2021). In this study, aiming to enhance the accuracy and efficiency of prognostic biomarkers, we first constructed a risk signature by pairing two irlnRNAs to evaluate the prognosis of BRCA patients, which only required determining the expression level of the irlncRNA pair. This method has the potential to reduce errors caused by different expression levels of lncRNAs, making our risk signature more practical and reliable.

We chose lncRNA MIR4435-2HG and lncRNA U62317.1 through the LASSO Cox regression analysis and the Kaplan-Meier survival analysis to establish a risk signature, where the two lncRNAs exhibited similar expression properties in BRCA patients. A higher expression of lncRNA U62317.1 compared to lncRNA MIR4435-2HG was defined as the low-risk group; otherwise, it was defined as the high-risk group. It has been revealed that lncRNA MIR4435-2HG may be a potential biomarker for the treatment and prognosis of liver cancer by upregulating the expression of B3GNT5 (Zhu et al., 2022) and may be a novel therapeutic target in gastric carcinoma by targeting the miR-138-5p/Sox4 axis (Gao et al., 2021).

Accurate prognostic assessment is essential for tumor treatment. After performing the Kaplan-Meier survival analysis, it was verified that the survival time of patients in the high-risk group was notably shorter than that of the patients in the low-risk group. In addition, it was confirmed that the scores of the DEirlncRNA pair and the clinicopathological characteristics were both associated with the prognosis of BRCA patients through univariate Cox regression analysis, as well as metastasis status was an independent predictor, and the DEirlncRNA pair as a prognostic marker could also independently predict the prognosis of BRCA patients through multivariate Cox regression analysis. Therefore, we convinced that our risk signature had significant prognostic value for BRCA patients.

Immune characteristics are closely associated with tumor progression and may influence the efficiency of tumor treatment by regulating the tumor immune microenvironment. Spearman correlation analysis was conducted to assess the data based on seven techniques, including XCELL, TIMER, QUANTISEQ, MCPCOUNTER, EPIC, CIBERSORT-ABS, and CIBERSORT, which indicated that the high-risk patients had lower immune and microenvironment scores. Previous studies have shown that immune and microenvironment scores are credible predictors of patient immunotherapy (Xu et al., 2020; Zou et al., 2020). After performing the Wilcoxon signed-rank test, we found that the high-risk group was negatively correlated with tumor-infiltrating immune cells, such as neutrophils, M1 macrophages, plasmacytoid dendritic cells, CD4^+^ T cells, and CD8^+^ T cells, whereas it was positively correlated with cancer-associated fibroblasts and M2 macrophages. Similarly, it has been reported that the abundance of infiltration of CD4^+^ T and CD8^+^ T cells plays an important role in immunotherapy by enhancing the immune response to tumor cells (Rodriguez Messan et al., 2021). Moreover, the high infiltration of M1 macrophages and the low infiltration of M2 macrophages predict satisfactory outcomes for tumor patients, because M1 macrophages have the ability to engulf cancer cells and M2 macrophages may inhibit inflammatory response and repair tissue (Al Haq et al., 2022). These studies provide theoretical support for our findings. In addition, ICs have the potential to be therapeutic targets for targeted immunotherapy. It has been reported that antibodies targeting CTLA-4, PD-L1, and PD-1 are ICB drugs used in the treatment of various malignancies (Archilla-Ortega et al., 2022). Thus, we assessed the correlation between the risk signature and the expressions of several ICs and discovered that the high-risk patients exhibited lower expressions of CD27, IDO1, and PCDC1, and higher expressions of CD276, which could be used in individual targeted immunotherapy of BRCA patients. It has been investigated that the CD27 intercellular domain could improve the chimeric antigen receptor targeting trophoblast cell surface antigen 2 (T2-CAR) T cell killing effect via multiple mechanisms (Chen H. et al., 2021) and targeting CD276 might reduce cancer stem cells (CSCs) in neck squamous cell carcinoma (HNSCC) through immune escape (Wang C. et al., 2021). Therefore, we confirmed that our risk signature might be used to select a more suitable immunotherapy method and could further predict the immunotherapy efficacy for BRCA patients.

Signaling pathways are involved in many critical biological processes, including proliferation, differentiation, apoptosis, and immune regulation of tumor cells, and they are strongly related to the occurrence, development, and treatment of tumors. After performing the Kaplan-Meier survival analysis and the univariate and multivariate Cox regression analyses successively, we recognized that only patients with higher expression of lncRNA MIR4435-2HG had a shorter survival time, and lncRNA MIR4435-2HG was identified as an independent predictor for BRCA patients. Thus, the high expression group with the expression levels of lncRNA MIR4435-2HG in the top 50% and the low expression group were subjected to GSEA to find that the high expression group was primarily enriched in some signaling pathways, such as KEGG-Proteasome, KEGG-EMC RECEPTOR INTERACTION, HALLMARK EMT, HALLMARK ANGIOGENESIS, and GOBP_REGULATION_OF_EXTRACELLULAR_MATRIX_ORGANIZATION. Some of these enriched pathways have been investigated in previous studies. For instance, it has been verified that the EMC-receptor interaction signaling pathway could regulate diverse cellular functions and is vital for sustaining normal homeostasis (Theocharis et al., 2016), and the angiogenesis signaling pathway could regulate the process of novel blood vessel development and is concerned with the growth, progression, and metastasis of tumors (Harry and Ormiston, 2021). It has also been suggested that the inhibition of angiogenesis and EMC may reduce the aggressiveness of tumors (Lima et al., 2019). These findings supported our idea that the blocking of these signaling pathways could be used to treat BRCA. Therefore, we determined that our risk signature had the capacity to provide downstream targets for the accurate treatment of BRCA patients.

Small-molecule drugs may show therapeutic potential, when tumor cells become resistant to conventional chemotherapeutic drugs and immune-targeted drugs. It has been proven that CHIR-99021 and FFAR1 modulator one are beneficial for tumor treatments. For instance, CHIR-99021, as an inhibitor of glycogen synthase kinase 3 (GSK3), exhibited antitumor activity in Merkel cell carcinoma (Houben et al., 2022); FFAR1 modulator 1 may activate the Hippo pathway to mediate apoptosis in androgen-independent prostate cancer cells (Wang et al., 2018). Therefore, we believed that these two small-molecular compounds would facilitate efficacy in the clinical treatment of BRCA patients.

On the whole, we constructed a prognostic risk signature with a lot of merit, but it still has several limitations. It is imperative for us to validate our risk signature by other independent cohorts owing to the high heterogeneity of BRCA. And we did not investigate our risk signature in cell experiments. However, we had verified its excellent values of survival time, clinicopathological characteristics, tumor-infiltrating immune cells, ICs, signaling pathways, and potential small-molecule drugs, and that made our signature remain reliable. In the future, our team will further investigate and validate the risk signature with more data and a larger clinical sample size.

## Conclusion

Our research exhibited that a novel signature independent of specific irlncRNAs expression was established by pairing two irlnRNAs to evaluate the prognosis value of BRCA patients and might be beneficial to selecting a more suitable and accurate immunotherapy method for the individual treatment of BRCA patients. We identified that this signature was closely associated with the prognosis and tumor immune microenvironment in BRCA patients, which had the potential to function as an independent prognostic biomarker and a predictor of immunotherapy in BRCA patients.

## Data Availability

The original contributions presented in the study are included in the article/Supplementary Material, further inquiries can be directed to the corresponding author.
