# Supplementary material for: An Immune-Related Long Noncoding RNA Pair as a New Biomarker to Predict the Prognosis of Patients in Breast Cancer
Source: Front Genet. 2022 Jun 22;13:895200. doi: 10.3389/fgene.2022.895200 (PMC9257047; doi:10.3389/fgene.2022.895200)
Supplement: Supplementary file 3 [file Table1.docx]

| Gene | HR | HR.95L | HR.95H | *p*-value |
| --- | --- | --- | --- | --- |
| AL645608.7\|LINC02446 | 1.644154 | 1.148213 | 2.354305 | 0.006638 |
| AL645608.7\|LINC02613 | 1.604699 | 1.140394 | 2.258043 | 0.006651 |
| AL645608.7\|NR4A1AS | 1.604455 | 1.127883 | 2.282396 | 0.008559 |
| AL645608.7\|SIAH2-AS1 | 1.546006 | 1.119458 | 2.135081 | 0.008167 |
| AL645608.7\|U62317.1 | 1.753076 | 1.236491 | 2.485481 | 0.001623 |
| LINC02613\|TFAP2A-AS1 | 0.672802 | 0.469044 | 0.965075 | 0.031311 |
| MIR4435-2HG\|U62317.1 | 1.604153 | 1.11133 | 2.31552 | 0.011615 |
| NR4A1AS\|TFAP2A-AS1 | 0.688904 | 0.475341 | 0.998416 | 0.049029 |

Supplemental Table 1: DEirlncRNA pairs with prognostic value using univariate Cox regression analysis.
