# Supplementary figures and images for: An Immune-Related Long Noncoding RNA Pair as a New Biomarker to Predict the Prognosis of Patients in Breast Cancer
Source: Front Genet. 2022 Jun 22;13:895200. doi: 10.3389/fgene.2022.895200 (PMC9257047; doi:10.3389/fgene.2022.895200)

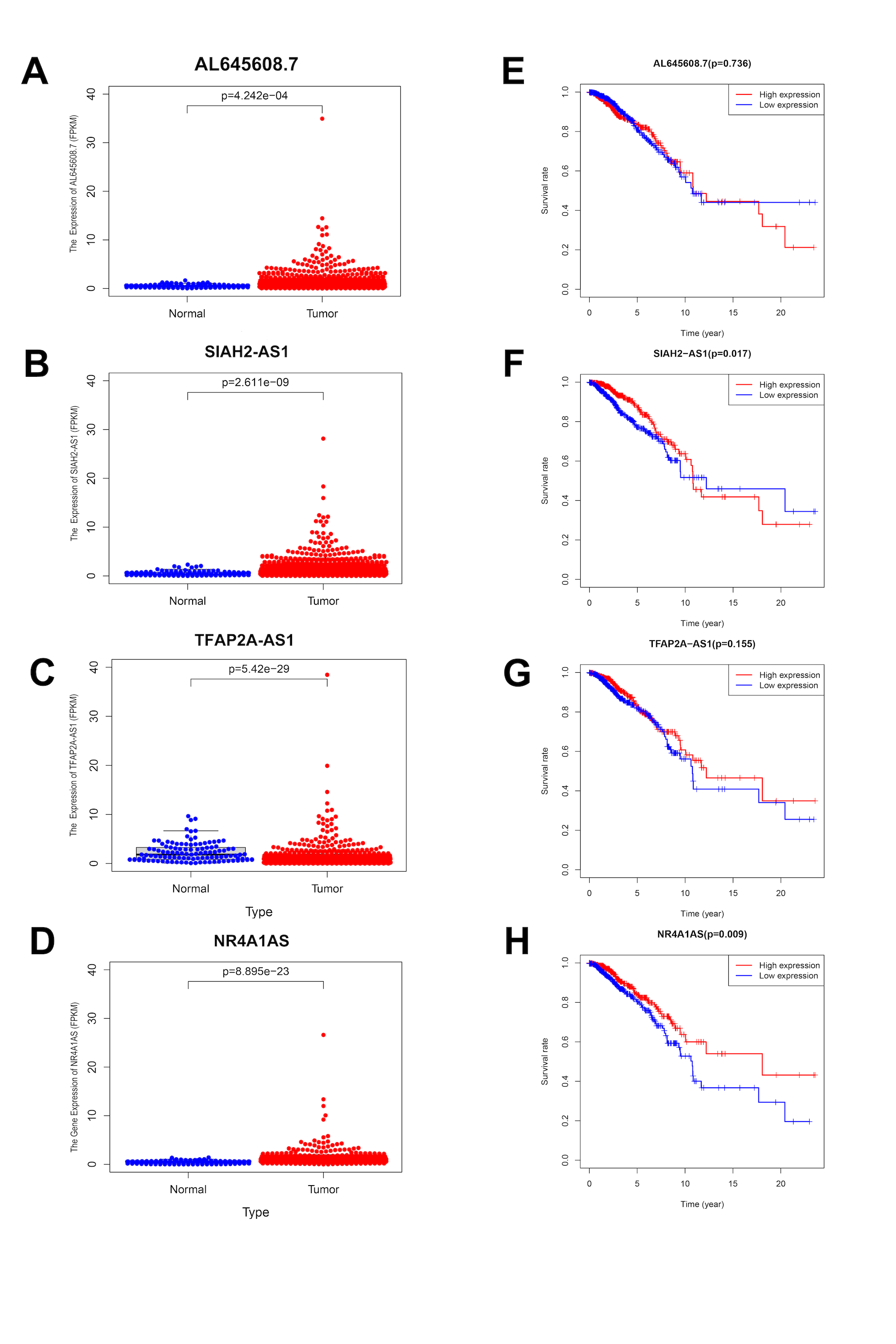

Supplement: Supplementary file 4 [file Image3.TIF]

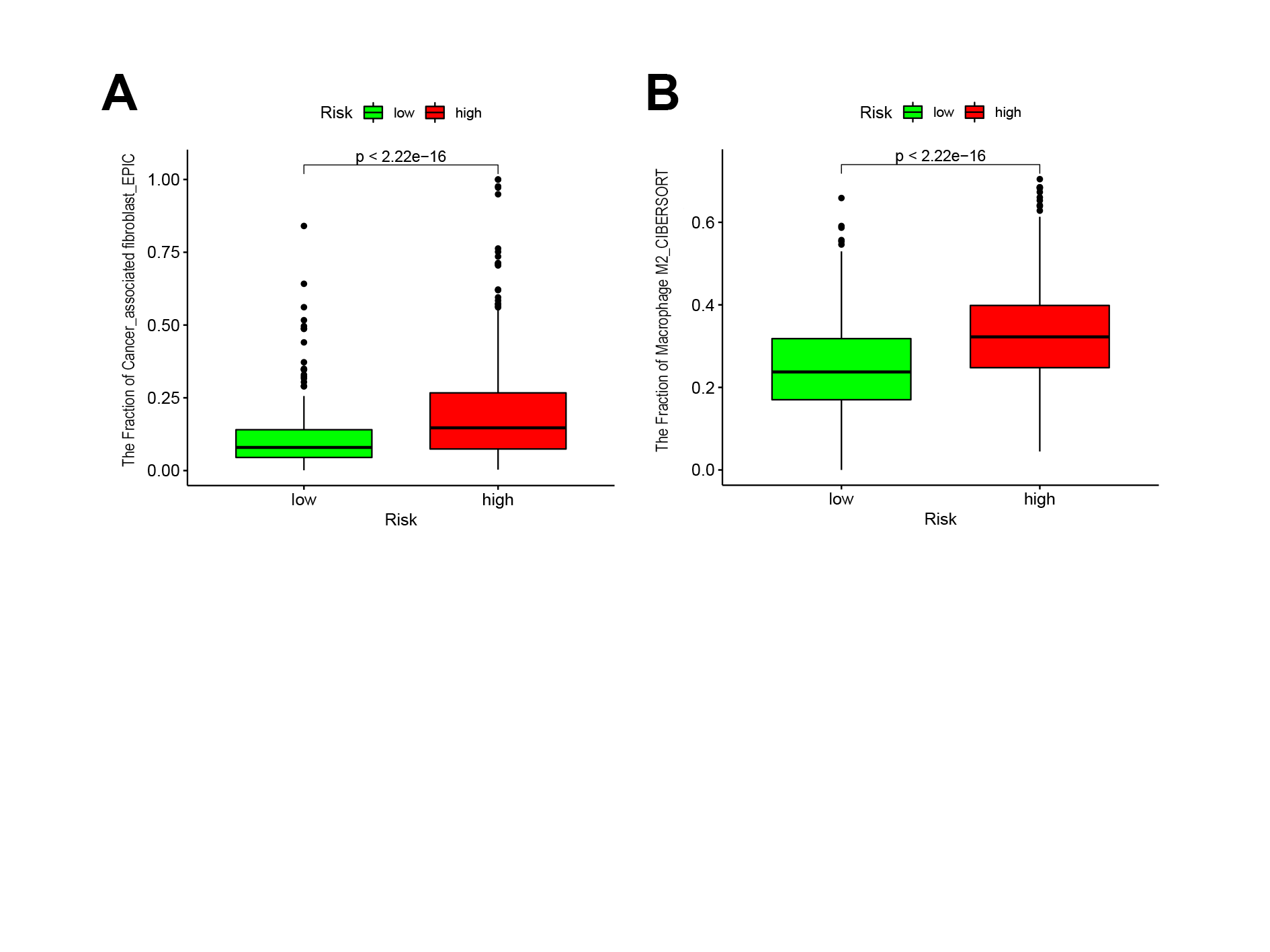

Supplement: Supplementary file 5 [file Image4.TIF]

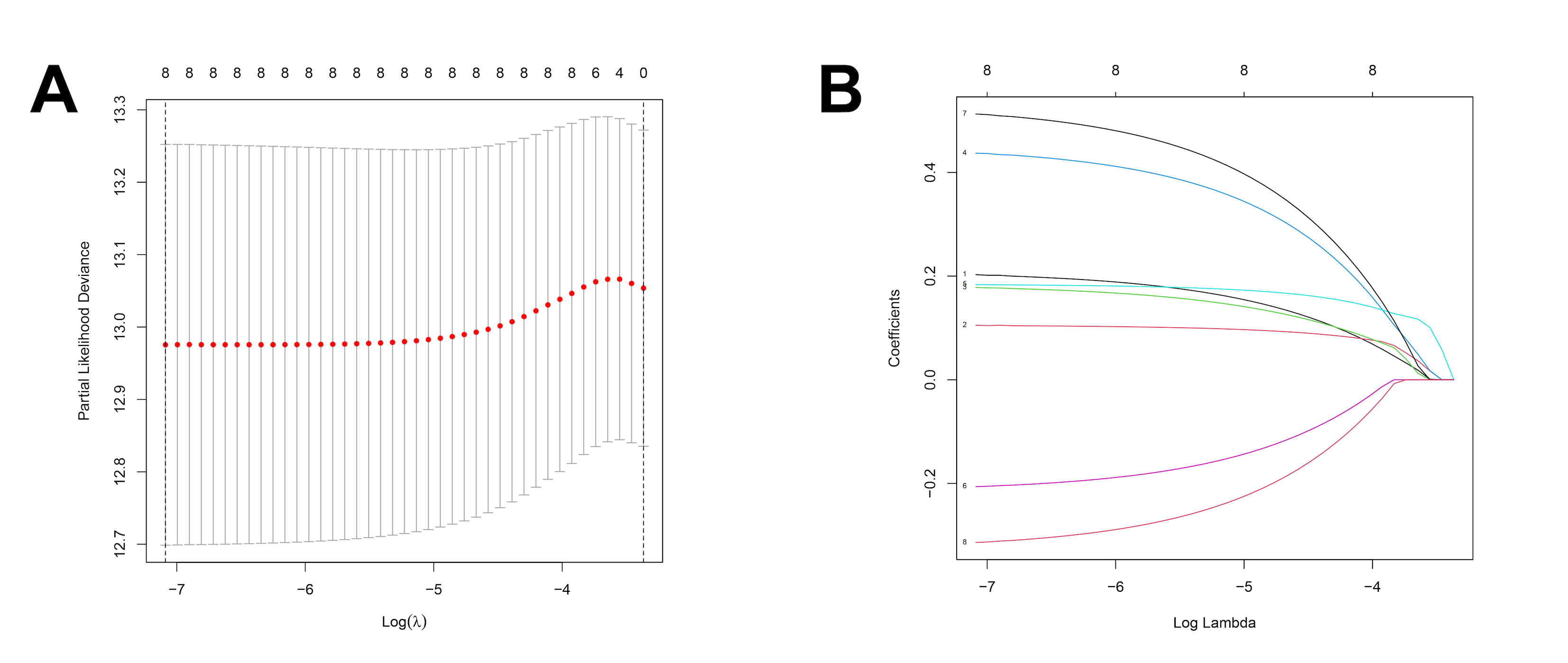

Supplement: Supplementary file 6 [file Image2.TIF]

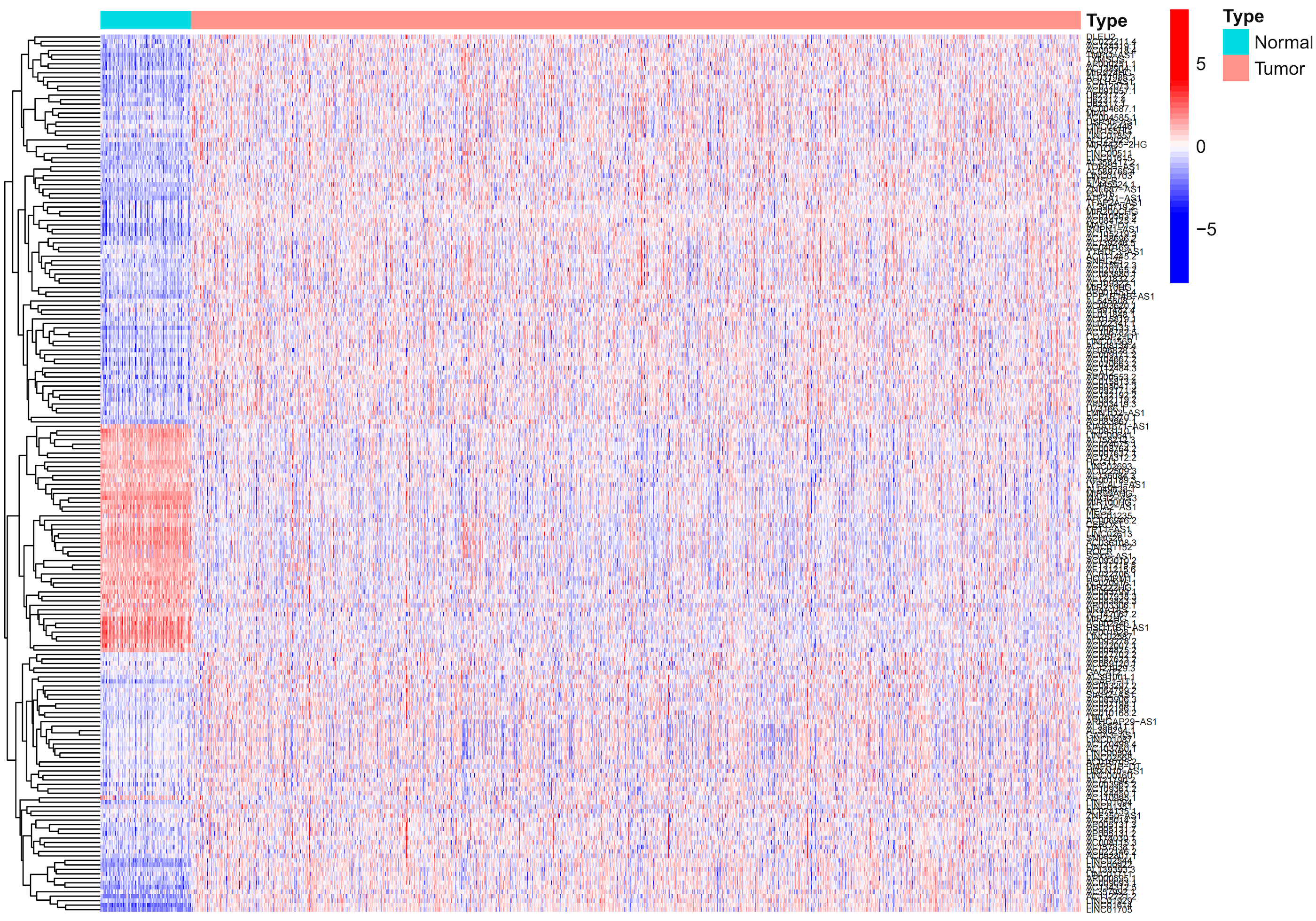

Supplement: Supplementary file 8 [file Image1.TIF]

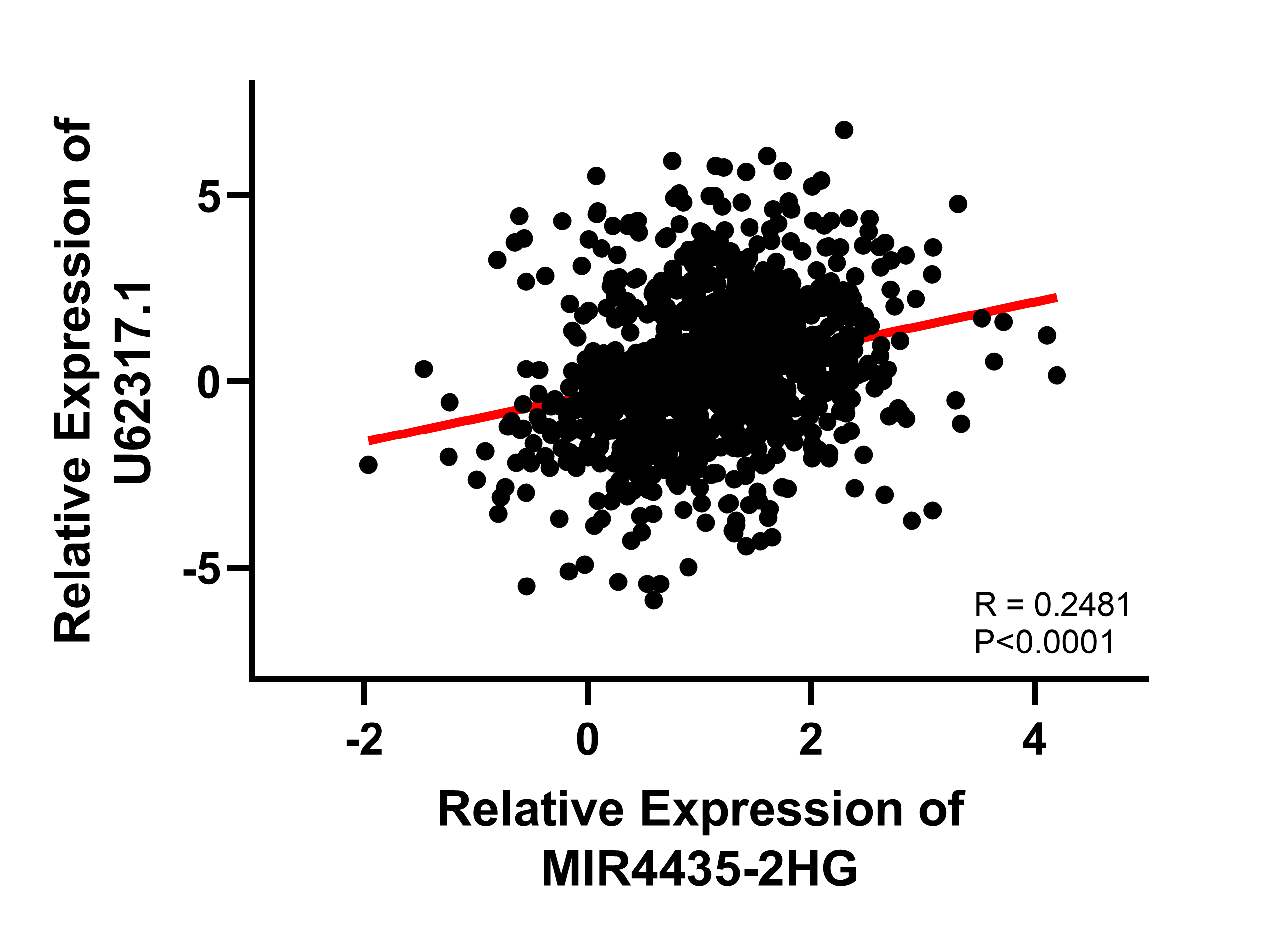

Supplement: Supplementary file 9 [file Image5.TIF]
